# Health-Promoting Behavior and Influencing Factors in Young North Korean Refugees (NKRs) Living in South Korea

**DOI:** 10.1007/s10903-018-0691-z

**Published:** 2018-02-02

**Authors:** Jumin Park, Young Dae Kwon, Hyunchun Park, Shi Eun Yu, Jin-Won Noh

**Affiliations:** 1 National Institutes of Health Clinical Center, Bethesda, MD USA; 2 Department of Humanities and Social Medicine, College of Medicine and Catholic Institute for Healthcare Management, the Catholic University of Korea, Seoul, South Korea; 3 Department of Healthcare Management, Eulji University, Seongnam, South Korea; 4 Clinical Trial Center, Korea University Medical Center, Seoul, South Korea; 5 Department of Healthcare Management and Institute of Global Healthcare Research, Eulji University, 553, Sanseong-daero, Sujeong-gu, Seongnam-si, Gyeonggi-do 13135 South Korea

**Keywords:** North Korea, Refugee, Health-promoting lifestyle behavior, Youth

## Abstract

The number of young North Korean refugees (NKRs) entering South Korea to escape famine and poverty and improve their quality of life is drastically increasing. The aims of this study were to identify and compare health promoting lifestyle behaviors (HPLBs) of young NKRs, compared to South Koreans, and to investigate influencing factors related to HPLBs in young NKRs. Data were obtained from 150 NKRs residing in South Korea and 161 South Koreans. Respondents provided their psychological status (depression, stress, and life satisfaction) and HPLBs. The NKRs reported lower interpersonal relations scores and higher spiritual growth scores compared to the control group. Attendance in religious services, stress, and life satisfaction were significantly associated with HPLBs in young NKRs. Health education and/or promotion programs focusing interpersonal relations and spiritual growth may be beneficial. In addition, regular psychological health screening is proposed as part of health-checkup programs, potentially improving adjustment to South Korean society.

## Background

The number of North Korean refugees (NKRs) entering into South Korea has drastically increased over the last decade as many look to escape from poverty and food shortages resulting from inefficient economic infrastructure and famine in North Korea [1–3]. The total number of NKRs entered South Korea in 2009 was estimated to be 2914, while it was only 71 in 1998. The number of NKRs sharply increased since 2002, bringing the total cumulative NKRs to 29,830 in September 2016. As more refugee families flee North Korea, the number of young NKRs has also increased: the number of adolescents and young adults (age 10–29 years) is approximately 12,000 in 2016, accounting for about 40% of the total NKRs [4]. Along with the increase in young NKRs, the successful adjustment of refugees to South Korean society has become an important concern [2].

For refugees, such as NKRs, living in a new environment, it is likely to be challenging to engage in health-promoting lifestyle behaviors (HPLBs), activities that an individual chooses to help achieve or maintain their health and well-being within various environments [5]. Lack of engagement in HPLBs is a significant risk factor for overall health status and chronic health conditions [6]. NKRs have been reported to have a worse health and a higher rate of illness compared to South-Koreans due to malnutrition, poor and unhealthy environment, and the defective healthcare system in North Korea [7, 8]. Their physical health also has been deteriorating when they roamed around in China and other neighboring countries before re-settlement in South Korea [7, 9].

A healthy life is the foundation for refugees to successfully adapt to their new lives. The change in health behaviors over time can contribute to the overall health status of refugees [10]. Accordingly, health promoting behaviors in young NKRs are crucial in successful adjustment into South Korean society. It indicates the need for an understanding of HPLBs and its influencing factors of young NKRs. There have been few studies on the evaluation of HPLBs in the young NKRs. In addition, NKRs are a unique immigrant group sharing the same genetic attributes and language as South Koreans, but have been exposed to two different environments while ordinary migration studies explore a group that migrated to different nations, this study uniquely looks at migrants within a recently divided nation. The purposes of the study were to identify HPLBs of young NKRs, compared to young South Koreans, and to investigate influencing factors related to HPLBs in young NKRs.

## Methods

### Design, Participants, and Data Collection

A cross-sectional survey design was used. The subjects of this study were 150 NKRs residing in South Korea as the refugee group, and 161 South Korean youth as the control group in the same region. NKRs were recruited by convenience sampling from alternative schools, local Hana Centers, and local social welfare organizations, between December 2013 and February 2014. Three NKR participants completed a pre-survey evaluation, which allowed us to confirm that they clearly understood the questions in Korean. 153 subjects agreed to the survey and a total of 150 NKRs were included after excluding the three subjects who participated in the pre-survey evaluation. The subjects in the control group, matched for age and sex with their NKR counterparts, were recruited by convenience and snowball sampling from middle schools, high schools, and colleges between August 2014 and October 2014. 162 subjects agreed to take part in the study and 161 were included after excluding one subject with incomplete response. The study has been performed with the approval of the Internal Review Board at Eulji University (EU 13-36).

### Measures

Socio-demographic variables were age, sex, religion, number of years settled in South Korea, presence of parents or relatives in South Korea, and presence of parents or relatives in North Korea. Regular diet was assessed on a three-point scale (*always, sometimes, rarely*) by asking, “Do you eat regularly?” Regular diet was considered to mean three meals (breakfast, lunch, and dinner) per day. Self-rated health status was measured on a five-point scale (*very well, well, normal, bad, very bad*) by asking, “What do you think about your health?”

Psychological status included stress, depressive symptoms, and life satisfaction. Stress was assessed by a 16 item questionnaire. The stress questionnaire was based on Daily Hassles Questionnaire by Rowlison and Felner [11] and revised by Shim [12] for students in South Korea. Items were scored using a five-point Likert scale from 1 (*not at all*) to 5 (*very much*), with a higher score indicating higher stress. The Cronbach’s alpha coefficient was 0.85. Depressive symptoms were assessed by the Center for Epidemiologic Studies Short Depression Scale (CES-D 10) [13]. This is a 10 item self-report measure with a four-point rating scale (0–3), ranging from 0 to 30. Participants were asked how frequently they experience depressive symptoms during the past week including four possible answers: 0 = *rarely* (less than 1 day); 1 = *sometimes* (from 1 to 2 days); 2 = *often* (from 3 to 4 days); and 3 = *at all time* (from 5 to 7 days). The internal consistency of CES-D 10 was demonstrated by a Cronbach’s alpha coefficient of 0.82. The Satisfaction with Life Scale [14] was used to measure global cognitive judgments of satisfaction with one’s life. This is a five-item questionnaire using a seven-point Likert scale from 1 (*strongly disagree*) to 7 (*strongly agree*), ranging from 5 to 35. The Cronbach’s alpha coefficient was 0.78.

HPLBs were assessed using a 52 item of the Health Promoting Lifestyle Profile II (HPLP-II), developed by Walker et al. [15]. The previously translated and validated Korean versions of the HPLP-II was used to measure HPLBs [16, 17]. The self-reported questionnaire included six subscales: health responsibility (nine items), physical activity (eight items), nutrition (nine items), spiritual growth (nine items), interpersonal relations (nine items), and stress management (eight items). Items were scored using a four-point Likert scale from 1 (*never*) to 4 (*routinely*). This measure has been shown to have validity and reliability in Koreans, including Cronbach alpha of 0.93 in Kim et al.’s study [16] and 0.93 in Yun and Kim’s study [17]. In this study, the internal consistency of the instrument was acceptable (Cronbach alpha for instrument as a whole = 0.91; for nutrition = 0.81; for physical activity = 0.82; for stress management = 0.71; for spiritual growth = 0.81; for health responsibility = 0.71; and for interpersonal relations = 0.75).

### Analysis

Descriptive statistics were used to show the distribution of socio-demographic and psychological variables. Differences between the NKRs and South Korean residents were tested by analysis of covariance (ANCOVA) corrected according to the age and sex of the subjects. Socio-demographic factors (age, sex, religion, number of years settled in South Korea, presence of parents or relatives in South Korea, and presence of parents or relatives in North Korea) and psychological status (stress, depressive symptoms, and life satisfaction) were included in the bivariate analyses. Factors related to HPLB subscales at *p* < 0.10 were included in the multiple linear regression models to evaluate the potential predictors to HPLB subscales. A priori known factors age and sex were controlled in the model. Finally, a multiple regression analysis was conducted to determine significant predictors for HPLB subscales. All the statistical analyses were performed with IBM SPSS software package version 22.0 [18].

## Results

The study population consisted of 150 NKRs (age 12–27 years) and 161 South Korean residents (age 13–27 years). The NKRs were predominately female (57.8%) and reported religious affiliation as Christian (59.2%). The majority of the South Korean controls were female (60.5%) and more than half indicated no religion affiliation (54.1%). The NKRs group reported having less regular meals than the South Korean Controls (*p* = 0.019). There was no significant difference in self-rated health status between the two groups. In the NKRs, a number of years settled in South Korea was under 3 years in 69.4%. 73.8% of young NKRs reported having parents or relatives in South Korea and 74.8% of young NKRs have parents or relatives in North Korea (Table 1).

**Table 1:** Participants’ characteristics

Variables	n (%)		*p*-Value
	NKRs (N = 150)	SK controls (N = 161)	
Age, mean (SD), year	21.43 (2.9)	20.10 (3.1)	< 0.001*
Range	12–27	13–27	
Sex			0.634
Male	62 (42.2)	62 (39.5)	
Female	85 (57.8)	95 (60.5)	
Religion			< 0.001*
Christian	87 (59.2)	51 (32.1)	
Buddhist	4 (2.7)	4 (2.5)	
Catholic	1 (0.7)	18 (11.3)	
Other	2 (1.4)	0 (0.0)	
None	53 (36.0)	86 (54.1)	
Regular diet			0.019*
Always	35 (24.0)	61 (38.1)	
Sometimes	71 (48.6)	69 (43.1)	
Rarely	40 (27.4)	30 (18.8)	
Self-rated health status			0.931
Very well	14 (9.5)	19 (12.0)	
Well	56 (38.1)	64 (40.3)	
Normal	60 (40.8)	60 (37.7)	
Bad	16 (10.9)	15 (9.4)	
Very bad	1 (0.7)	1 (0.6)	
Number of years settled in South Korea			
Under 3 years	75 (69.4)	N/A	
More than 3 years	33 (30.6)	N/A	
Parents or relatives in South Korea			
Yes	93 (73.8)	N/A	
No	33 (26.2)	N/A	
Parents or relatives in North Korea			
Yes	95 (74.8)	N/A	
No	32 (25.2)	N/A	

*NK* North Korea, *SD* standard deviation, *SK* South Korea, *N*/*A* not available

*p < 0.05

Table 2 presents the means and standard errors for the scores for overall HPLB and each of the six subscales of the HPLBs for both groups. After adjusting for age and sex, overall HPLB score between NKRs and South Korean controls was not significantly different, *F* (1, 270) = 3.135, *p* = 0.301. NKRs did however show significantly higher scores of spiritual growth (*F* (1, 261) = 7.829, *p* = 0.006) and lower scores of interpersonal relations (*F* (1, 264) = 10.051, *p* = 0.002) in HPLBs compared to South Korean controls. There was a significant difference in stress between the two groups (*p* = 0.007), but not in life satisfaction and depressive symptoms (*p* > 0.05) (Table 2).

**Table 2:** Comparisons of HPLBs and psychological status between NKRs and SK controls

Variables	Mean (SE)		*p*-Value
	NKRs (N = 150)	SK controls (N = 161)	
Health-promoting behavior	2.48 (0.03)	2.53 (0.03)	0.301
Health responsibility	2.19 (0.05)	2.16 (0.05)	0.608
Physical activity	2.30 (0.05)	2.27 (0.05)	0.627
Nutrition	2.45 (0.04)	2.53 (0.04)	0.144
Spiritual growth	2.83 (0.05)	2.64 (0.05)	0.006*
Interpersonal relations	2.51 (0.04)	2.68 (0.04)	0.002*
Stress management	2.42 (0.04)	2.74 (0.03)	0.078
Psychological status			
Stress	45.24 (1.00)	49.00 (0.95)	0.007*
Depressive symptoms	10.85 (0.47)	9.82 (0.45)	0.116
Life satisfaction	20.69 (0.50)	19.93 (0.48)	0.277

*NKRs* North Korea refugees, *SE* standard error, *SK* South Korea

*p < 0.05, ANCOVA adjusted for age and sex

### Predictors of HLPB Subscales

Table 3 presents the final multiple linear regression models for each outcome with age and sex as covariates in all models.

**Table 3:** Socio-demographic and psychological predictors of HPLBs in NKRs (N = 150)

Variables	Health responsibility		Physical activity		Nutrition		Spiritual growth		Interpersonal relations		Stress management	
	*B* (*SE*)	95% CI	*B* (*SE*)	95% CI	*B* (*SE*)	95% CI	*B* (*SE*)	95% CI	*B* (*SE*)	95% CI	*B* (*SE*)	95% CI
Age	0.012 (0.108)	(− 0.199, 0.453)	− 0.140 (0.129)	(− 0.466, 0.045)	− 0.113 (0.106)	(− 0.342, 0.087)	0.079 (0.123)	(− 0.120, 0.366)	0.107 (0.100)	(− 0.077, 0.321)	− 0.129 (0.103)	(− 0.347, 0.059)
Sex (female)	0.058 (0.709)	(− 0.944, 1.869)	− **0.301 (0.842)**^‡^	(− 4.628, − 1.293)	0.087 (0.688)	(− 0.705, 2.023)	0.037 (799)	(− 1.222, 1.950)	0.140 (0.673)	(0.311, 2.359)	− 0.049 (0.662)	(− 1.660, 0.966)
Religion	**0.180 (0.757)** ^†^	(0.015, 3.015)	–	–	–	–	**0.275 (0.834)** ^‡^	(1.170, 4.480)	**0.189 (0.694)** ^‡^	(0.062, 2.817)	–	–
Parents or relatives in SK	–	–	–	–	–	–	–	–	0.133 (0.661)	(− 0.334, 2.289)	–	–
Parents or relatives in NK	− 0.123 (0.763)	(− 2.573, 0.453)	–	–	–	–	–	–	–	–	–	–
Stress	− **0.181 (0.029)**^†^	(− 0.199, − 0.003)	− **0.186 (0.035)**^†^	(− 0.145, − 0.007)	− 0.130 (0.029)	(− 0.098, 0.016)	− **0.191 (0.070)**^†^	(− 0.150, − 0.014)	− 0.159 (0.028)	(− 0.108, 0.005)	− 0.171 (0.028)	(− 0.109, 0.004)
Depressive symptoms		–	–	–	–	–	− 0.080 (0.077)	(− 0.227, 0.080)	− 0.061 (0.066)	(− 0.173, 0.087)	− 0.026 (0.065)	(− 0.148, 0.112)
Life satisfaction	**0.297 (0.062)** ^‡^	(0.086, 0.331)	–	–	**0.219 (0.062)** ^†^	(0.027, 0.274)	0.478 (0.070)*	(0.268, 0.547)	**0.316 (0.059)** ^‡^	(0.089, 0.322)	**0.276 (0.060)** ^‡^	(0.056, 0.293)
*R*^2^	0.215		0.153		0.093		0.420		0.266		0.143	
*F*	4.836*		6.993*		2.858^†^		44.585*		5.067^†^		3.481^‡^	

*CI* confidence interval, *NK* North Korea, *SK* South Korea, *SE* standard error

Sex coded as 0 = male, 1 = female; Religion coded 0 = no, 1 = yes; Parents or relatives in South Korea coded 0 = no, 1 = yes; Parents or relatives in North Korea coded 0 = no, 1 = yes

*p < 0.001; ^†^*p* < 0.05; ^‡^*p* < 0.01, bold represents the significant *p*-values

#### Health Responsibility

Four factors (religion, parents or relatives in North Korea, stress, life satisfaction) met the bivariate analyses criteria and were included in a standard multiple linear regression model predicting health responsibility. The final model was significant, *F* (6, 106) = 4.836, *p* < 0.001, and accounted for 21.5% of the variance in health responsibility. Participants who attended religious activities (*β* = 0.180, *p* = 0.048), and experiencing low levels of stress (*β* = − 0.181, *p* = 0.041) and high levels of life satisfaction (*β* = 0.297, *p* = 0.001) were more likely to take greater responsibility for their medical health.

#### Physical Activity

Stress was the only factor to meet the bivariate association criteria for physical activity. The final regression model including age, sex, and stress was significant and the total variance explained by the model was 15.3% (*F* (3,116) = 6.993, *p* < 0.001). Females (*β* = − 0.301, *p* = 0.001), and respondents experiencing high levels of stress (*β* = − 0.186, *p* = 0.032) were less likely to exercise.

#### Nutrition

Two factors (stress, life satisfaction) met the bivariate association criteria with nutrition in HPLBs and were entered in a final regression model. The final model was significant, *F* (4,111) = 2.858, *p* = 0.027, and accounted for 9.3% of the variance in nutrition. Adjusted multiple regression models revealed that life satisfaction was positively associated with nutrition in HPLBs (*β* = 0.219, *p* = 0.017).

#### Spiritual Growth

Four factors (religion, stress, life satisfaction, depressive symptoms) met the bivariate analyses criteria. The final regression model was significant and the total variance explained by the model was 42.0% (*F* (6, 96) = 11.585, *p* < 0.001). Respondents who attended religious activities (*β* = 0.275, *p* = 0.001), and experiencing low levels of stress (*β* = − 0.191, *p* = 0.019) and high levels of life satisfaction (*β* = 0.478, *p* < 0.001) were more likely to report higher scores of spiritual growth in HPLBs.

#### Interpersonal Relations

Five factors (religion, parents or relatives in South Korea, stress, depressive symptoms, life satisfaction) met the bivariate analyses criteria and were entered in a final regression model. The final model was significant, *F* (5,104) = 3.481, *p* = 0.006, and accounted for 14.3% of the variance in interpersonal relations. Participants who attended religious activities (*β* = 0.189, *p* = 0.041), and experiencing high levels of life satisfaction (*β* = 0.316, *p* = 0.001) were more likely to take greater interpersonal relations in HPLBs.

#### Stress Management

Three factors (stress, depressive symptoms, life satisfaction) met the bivariate analyses criteria. The final model including age, sex, stress, depressive symptoms, and life satisfaction was significant and the total variance explained by the model was 14.3% (*F* (5,104) = 3.481, *p* < 0.006). Participants with higher levels of life satisfaction had higher score of stress management in HPLBs (*β* = 0.276, *p* = 0.004).

## Discussion

This study is the first we know of to evaluate HPLBs in young NKRs as compared to South Korean residents. The average HPLB score of NKRs was 2.48 (range 1–4), somewhat higher than those of NKR adults over 20 years aged by Choe et al. [19] and Kang et al. [20] (Means = 2.38 and 1.78, respectively). Among six HPLB dimensions, health responsibilities and physical activity were the least frequent and spiritual growth and interpersonal relations were the most frequent in young NKRs. This is consistent with previous studies of HPLBs in adults of NKRs [19, 20]. The results demonstrated that young NKRs have better HPLB than adult NKRs, but similar patterns of health-promoting behaviors are shown across age groups in NKRs.

We predicted that there would be differences in HPLBs between the young NKRs and South Korean residents because the NKRs, having severe traumatic experiences and adjusting new environment, might find it more challenging to engage in HPLBs. We found that there were significant differences in interpersonal relations and spiritual growth of HPLBs between the two groups. Regarding personal relations in HPLBs, young NKR scored significantly lower than South Korean residents. The young NKRs may be used to a closed, communist society, and thus they may experience cultural conflict after entering South Korea [2, 21, 22]. Young NKRs are also often hesitant to speak in North Korean dialect to avoid revealing their background and identity in schools or workplaces [21, 22]. Given the cultural conflict and language barrier for young NKRs, they often experience difficulties in personal communications. Moreover, since more than half a century has passed since the division of the two Koreas, the younger generation of South Koreans are less likely to consider North Koreans as part of the same nation and may have negative attitudes toward them, compared to the sixty-or-older group [1, 22]. For example, young NKRs report having experienced social discrimination by peers or co-workers [22]. As a result, many young NKRs feel estranged from the South Korean society, and their South Korean peers find it difficult to get along with them [1, 22, 23].

Of note in our study, the HPLBs for spiritual growth in young NKRs was higher than South Korean residents. In addition, significantly more respondents in the NKR group (64.0%) reported practicing a religion, mainly Christianity, than the South Korean controls (45.9%). During the journey from North to South Korea, many religion-based organizations, mostly Christian, help refugees to adjust during the migration process and some refugees become Christians while staying in China [2]. Indeed, churches and Christian organizations play an important role in the lives of NKRs in South Korea [24, 25]. Religious services have been shown to help them better adjust by offering information regarding living in a new environment, and by providing a place where the individual can feel a sense of belonging and gain support by sharing their experiences, feelings, and thoughts [25]. The churches and Christian organizations might be effective in assisting the NKRs in their adjustment process to living in South Korea.

The study also analyzed the socio-demographic and psychological factors associated with HPLBs. Attendance in religious services, stress, and life satisfaction significantly were associated with HPLBs in young NKRs. NKRs who attended religious activities reported significantly higher levels of health responsibility, spiritual growth, and interpersonal relations in HPLBs. The observation is consistent with previous research demonstrating religious involvement significantly promoting HPLBs, which in turn benefit health and prolong life [26–29]. Greater religious involvement was related to higher optimism and hope, greater meaning or purpose in life, and health [28]. The active and optimistic attitude toward life may help explain the relationship between religion and HPLBs. Previous research reported that religious involvement was associated with greater use of preventive care services, such as regular medical checkups [26, 29]. Particularly Korean churches can serve many important roles for immigrants by facilitating and mobilizing positive interpersonal support and resource to a community [27]. The support received from their religious community likely functions similarly for NKRs living in South Korea.

Young NKRs with high levels of stress in this study reported poorer health responsibility, physical activities, and spiritual growth in HPLBs. In addition, young NKRs who present low level of life satisfaction had poorer in health responsibility, nutrition, spiritual growth, interpersonal relations, stress management in HPLBs. This is consistent with previous research indicating that poor HPLBs was predicted by high stress [30, 31] and low life satisfaction [32–34]. The relationship between psychological status and HPLBs suggest that particular attention should be given to those with a high level of stress and low level of life satisfaction. There is a need for regular psychological status screening and support programs to relieve stress and enhance life satisfaction in young NKRs for improving their HPLBs. Additionally, several studies found that HPLBs was predicted by socio-demographic factors, such as age [35], gender [35, 36], and living with their own family [34]. In contrast to previous results, socio-demographic factors did not relate to the decision to adopt HPLBs in this study. More studies are needed to understand the effect of socio-demographic factors on HPLBs in NKRs.

This study has several limitations. First, the convenience sample was not randomly selected, and therefore the subjects in this study may not be a representative sample of all young NKRs living in South Korea. However, opportunities to contact NKRs are limited and information collection is restricted because of their concerns about identity exposure, avoidance of external contact, and language barriers. Second, the design of this study was cross-sectional, which may hinder interpreting as a causal relationship. Further longitudinal design and mixed method approaches are needed to gain in-depth information about HPLBs in young NKRs.

## Conclusions

The findings of this study demonstrate that there are deficits in HPLBs particularly for young NKRs, specifically in the sub-domains of interpersonal relations. Indeed, religious involvement, levels of stress and life satisfaction are related to the young NKRs’ decision to adopt the HPLBs. In this regard, more emphasis should be directed toward encouraging the young NKRs to practice HPLBs by continuous integration of intervention programs and related health policies, especially focusing on interpersonal relations. In addition, regular psychological status screening is proposed as part of health-checkup programs.
